# Study protocol: the effectiveness and cost effectiveness of an employer-led intervention to increase walking during the daily commute: the Travel to Work randomised controlled trial

**DOI:** 10.1186/s12889-015-1464-4

**Published:** 2015-02-18

**Authors:** Suzanne Audrey, Ashley R Cooper, William Hollingworth, Chris Metcalfe, Sunita Procter, Adrian Davis, Rona Campbell, Fiona Gillison, Sarah E Rodgers

**Affiliations:** School of Social and Community Medicine, University of Bristol, Canynge Hall, Whatley Road, Bristol, BS8 2PS UK; Centre for Exercise Nutrition and Health Sciences, and National Institute for Health Research, Bristol Biomedical Research Unit in Nutrition Diet and Lifestyle, School for Policy Studies, University of Bristol, 8 Priory Road, Bristol, BS8 1TZ UK; School of Social and Community Medicine, and Bristol Randomised Trials Collaboration (BRTC), University of Bristol, Canynge Hall, 39 Whatley Road, Bristol, BS8 2PS UK; Adrian Davis Associates, 49 Monmouth Road, Bristol, BS7 8LF UK; Department for Health, University of Bath, Bath, BA2 7AY UK; Farr Institute, College of Medicine, Swansea University, Swansea, SA2 8PP UK

**Keywords:** Active travel, Walking, Workplace, Physical activity measurement, Randomised controlled trial

## Abstract

**Background:**

Physical inactivity increases the risk of many chronic diseases including coronary heart disease, type 2 diabetes and some cancers. It is recommended that adults should undertake at least 150 minutes of moderate intensity physical activity throughout the week but many adults do not achieve this. An opportunity for working adults to accumulate the recommended activity levels is through the daily commute.

**Methods:**

Employees will be recruited from workplaces in south-west England and south Wales. In the intervention arm, workplace Walk-to-Work promoters will be recruited and trained. Participating employees will receive Walk-to-Work materials and support will be provided through four contacts from the promoters over 10 weeks. Workplaces in the control arm will continue with their usual practice. The intervention will be evaluated by a cluster randomized controlled trial including economic and process evaluations. The primary outcome is daily minutes of moderate to vigorous physical activity (MVPA). Secondary outcomes are: overall physical activity; sedentary time; modal shift away from private car use during the commute; and physical activity/MVPA during the commute. Accelerometers, GPS receivers and travel diaries will be used at baseline and one year follow-up. Questionnaires will be used at baseline, immediately post intervention, and one year follow-up. The process evaluation will examine the context, delivery and response to the intervention from the perspectives of employers, Walk-to-Work promoters and employees using questionnaires, descriptive statistics, fieldnotes and interviews. A cost-consequence study will include employer, employee and health service costs and outcomes. Time and consumables used in implementing the intervention will be measured. Journey time, household commuting costs and expenses will be recorded using travel diaries to estimate costs to employees. Presenteeism, absenteeism, employee wellbeing and health service use will be recorded.

**Discussion:**

Compared with other forms of physical activity, walking is a popular, familiar and convenient, and the main option for increasing physical activity in sedentary populations. To our knowledge, this is the first full-scale randomised controlled trial to objectively measure (using accelerometers and GPS receivers) the effectiveness of a workplace intervention to promote walking during the commute to and from work.

**Trial registration:**

ISRCTN15009100 (10 December 2014).

## Background

Physical inactivity increases the risk of many chronic diseases including coronary heart disease, type 2 diabetes, obesity and some cancers [1]. It is currently recommended that adults should undertake at least 150 minutes of moderate intensity physical activity in bouts of 10 minutes or more throughout the week [1-3]. There are concerns that many adults in the United Kingdom and other high-income countries do not achieve this [1,3-5].

Increasing physical activity levels, particularly among the most inactive, is an important aim of current public health policy in the UK [1,6]. There is also increasing interest in the relationship between time spent sedentary and poor health outcomes [7] and consequently UK health guidelines recommend that adults should minimise the amount of time spent sedentary (sitting) in addition to increasing physical activity [1].

Adult obesity levels may be linked to travel behaviour, one indicator of which is that countries with highest levels of active travel tend to have the lowest obesity rates [8]. Walking is a carbon neutral mode of transport that has declined in recent decades in parallel with the growth in car use [1]. Walking has been described as the nearest activity to perfect exercise [9]. It is a popular, familiar, convenient and free form of exercise that can be incorporated into everyday life and sustained into older age. Even walking at a moderate pace of 5 km/h (3miles/h) expends sufficient energy to meet the definition of moderate intensity physical activity [10]. Hence there are compelling reasons to encourage people to walk more, not only to improve their health but to address the problems of climate change [11-14].

In the UK, there are substantial opportunities to increase walking by replacing short journeys undertaken by car. For example, the 2011 National Travel Survey showed 22% of all car trips were shorter than two miles in length, while 18% of trips of less than one mile were made by car [15]. An opportunity for working adults to accumulate the recommended moderate activity levels is through the daily commute and, in addition, replacing the car for short journeys is likely to reduce sedentary time. Experts in many World Health Organisation (WHO) countries agree that significant public health benefits can be realised through greater use of active transport modes [16]. Furthermore, cost benefit analysis for the UK Department for Transport suggests the ratio of benefits to costs are high [17].

A systematic review comparing direct versus self-report measures for assessing physical activity in adults found self-report measures provided were higher estimates of physical activity than objective measures in some cases and lower values in others [18]. This calls into question the reliability of self-report measures, and indicates there is no approach to correcting for self-report measures that will be valid in all cases. However, very few studies have objectively measured the contribution of walking, particularly walking to work, to adult physical activity levels and more evidence is needed [19].

Systematic reviews have examined the effectiveness of interventions to promote physical activity in general [20-23] but there is less evidence about how best to promote walking to work. Available systematic review evidence has focused on: interventions that promote walking; [24] interventions that promote walking and cycling as an alternative to car use [23], and; the effectiveness of workplace physical activity interventions [25]. NICE public health guidance on workplace health promotion concluded that although schemes exist to encourage employees to walk or cycle to work, little is known about their impact [26]. Few studies used robust data collection methods to measure the impact of workplace interventions on employees’ physical activity levels, with most using self-report. There was also a lack of information about how interventions are influenced by the size and type of workplace and the characteristics of employees.

In 2011 the National Institute for Health Research Public Health Research (NIHR-PHR) programme funded the Walk to Work feasibility study (Project 10/3001/04) which incorporated Phase I development of a behavioural intervention followed by a Phase II exploratory trial in 17 workplaces in Bristol [27,28]. The intervention was tested in small, medium-sized and large workplaces, used objective measures of physical activity, and included process evaluation and an assessment of costs. Results from the feasibility study demonstrated that the intervention and its evaluation were feasible and funding was granted for a full-scale cluster randomised controlled trial commencing 1 November 2014 for 33 months.

## Methods/Design

### Aim

The overall aim of the research is to examine the effectiveness and cost effectiveness of an employer-led scheme to increase walking during the commute.

### Study design and setting

The study is a multi-centre cluster randomised controlled trial in 84 workplaces in south-west England and south Wales, and incorporates process and economic evaluations. The intervention will be implemented in 42 workplaces; 42 workplaces will comprise the control arm.

### The intervention

Workplace ‘Walk-to-Work promoters’ will be identified (volunteers, or nominated by participating employers) and receive a half-day training package provided by public health and transport specialists about the benefits of walking during the commute and how to promote increased walking either by walking the entire route or mixing walking with other transport modes. The Walk-to-Work promoters will be given resource packs and trained to access relevant websites and toolkits. They will also be trained in the use of specific behaviour change techniques that form the basis of the intervention, including: providing information on the link between walking and health; prompting intention formation; identifying barriers to walking and ways to overcome them; prompting goal setting; prompting self-monitoring; identifying social support and encouragement; reviewing goals, and; relapse prevention. There is evidence that these techniques are effective in achieving behaviour change [29,30] and can be effectively delivered by non-specialists. Additional booklets will be provided for employers/managers with information and ideas of how the workplace can support increased walking during the daily commute.

Participating employees will be contacted by the Walk-to-Work promoter and given a Walk-to-Work pack including an information booklet, travel diary and pedometer. Goals for incorporating walking into the journey to and from work will be set. Further encouragement will be provided through four contacts from the Walk-to-Work promoter over the following 10 weeks (face-to-face, email or telephone as appropriate to the workplace size, resources and work routines). During this time the Walk-to-Work promoters will also be prompted and encouraged in their role by four email/telephone contacts from the research team.

### Assessment of harms

This is a low risk intervention and there were no reported adverse events during the feasibility study. However, participants will be encouraged to report adverse and serious adverse events. These may relate to road traffic injuries and collisions; personal safety of walkers; difficulties experienced by Walk-to-Work promoters, including disrupting usual working relationships and employers attitudes towards time taken out of usual work activities; and costs to employers, including disruption to work routines, of permitting the intervention during working hours. If adverse events are attributable to the intervention, other relevant participants will be informed immediately e.g. other employees taking a similar route. It is also possible that people with low activity and no history of walking will suffer initial muscle stiffness. In most cases this would be mild and is a normal consequence of increased physical activity. However, participants will be given information about symptoms which may require medical attention and temporary or permanent cessation of walking to work. Such incidents will be recorded and monitored throughout the trial.

### The control group

The control group will participate in the baseline and one year follow-up data collection activities. No promoters will be trained in the control workplaces but they may be exposed to other workplace, local or national walking initiatives during the intervention period. Information about involvement in such activities will be collected through the questionnaires at baseline, post-intervention and one year follow-up.

### Inclusion and exclusion criteria

Employees in participating workplaces in south-west England and south Wales will be eligible to take part. However, employees who already always walk or cycle to work, who are due to retire before the one year follow-up data collection, or who are disabled in relation to walking, will be excluded. Employees for whom daily driving is a key part of their role (for example, sales representatives) will also be excluded. Workplaces with a large proportion of staff on short-term or zero-hours contracts are not suited because a high proportion of these people will move on before the one-year follow-up data collection. In addition, workplaces with firm plans to significantly downsize or relocate during the study period will be excluded.

### Recruitment of workplaces

There will be two rounds of recruitment, during February and March 2015 and again in February to March 2016. Workplaces will be approached through the local Chambers of Commerce and other employer organisations, and will be sent basic information about the study and an invitation to participate.

### Allocation and randomisation

Randomisation will take place at the level of the workplace. Employers in workplaces expressing an interest will be asked to complete a short questionnaire to broadly ‘match’ pairs of workplaces with respect to: size (1–10, 50, 51–250 or 250+ employees); [31] location (urban or suburban), and; type of business (for example, office-based, manufacturing). Assignment of workplaces to the intervention group, within each matched pair, will employ concealed computer generated allocation by an independent researcher from Bristol Randomised Trials Collaboration to minimise selection bias. It is not possible to blind participants following randomisation for this intervention.

### Participant recruitment

Information and leaflets and invitations to participate in the study will be distributed to employees by the employers using their usual mode of contact, for example, by email, attachments to wage slips, in pigeonholes or during team meetings. This will vary by size and type of workplace and will be noted as part of the process evaluation.

### Primary and secondary outcomes

The primary outcome, measured at baseline and one year follow-up, is daily minutes of moderate to vigorous physical activity (MVPA), with secondary outcomes of overall levels of physical activity (counts per minute (cpm)), and modal shift (number of days, over the previous five working days, when walking was the major mode of travel to/from work). The process evaluation will focus on: facilitators and barriers to workplace/employer participation in walk to work interventions; facilitators and barriers to employees walking during the daily commute, and; physical activity/MVPA due to walking during the journey to/from work. The economic evaluation will examine: costs to employers and employees of implementing the Walk-to-Work scheme; consequences for the employer (absenteeism/presenteeism); consequences for employees (commuting costs and wellbeing), and consequences for the public sector (health service use).

### Assessment and follow-up

Before randomisation, participating employees will be asked to complete a baseline questionnaire giving basic personal data (sex, age, ethnicity), job title, mode of transport to work, before and after work ‘routines’ affecting travel mode (for example, taking young children to school), typical commuting costs, household car ownership, commute related adverse events, health service use, wellbeing (ICECAP-A [32],) and views about walking. They will also be asked to wear an accelerometer for seven days from waking in the morning until going to bed at night to provide an objective measurement of physical activity (including intensity), and to carry a personal global positioning system (GPS) receiver from leaving home until returning from work. Participants will also be asked to complete a travel diary, recording mode of travel, costs and journey times at baseline and one year follow-up.

Immediately after the 10-week intervention, questionnaires will be administered in intervention and control arms to explore: attitudes towards, and experiences of, walking during the daily commute including perceived barriers and facilitators, and emotional and physical well-being. Additional questions about the acceptability of the intervention will be included for the intervention arm only. At one year follow-up questionnaires, accelerometers and GPS receivers, and travel diaries will be administered in intervention and control arms (as per baseline protocol).

The costs of the intervention to employers will be assessed by recording all time spent on training the promoters, implementing the intervention among employees, and materials or resources used. Absenteeism will be monitored using both the self-reported workplace productivity and impairment (WPAI) questionnaire [33] and anonymized data from workplace human resource systems where available. Costs to participants will be assessed by recording journey time, household commuting costs and expenses at baseline and one year follow-up using travel diaries. Self-reported measures of health service use will allow us to provide preliminary evidence on the savings or costs to the wider society. Employee wellbeing will be measured using ICECAP-A [32], a recently developed broader measure of benefit for use in economic evaluations of population health and other interventions.

### Measuring physical activity

The accelerometer will generate measures of physical activity. GPS data will be time-matched with accelerometer data and visualised in a geographic information system (GIS). Journeys to and from work will be identified and the data segmented to provide a measure of duration of the journey to work and associated physical activity. Validated accelerometer thresholds will be used to compute daily time spent in MVPA and sedentary.

### Process evaluation

The process evaluation will examine the context, delivery and response to the intervention from the perspectives of employers, Walk-to-Work promoters and employees. Interviews will be conducted immediately post intervention with a purposive sample of employees who have increased walking during the commute (n = 18), and employees who have not (n = 18). The sample will be stratified by workplace characteristics and gender. A purposive sample of employers (n = 12) and Walk-to-Work promoters (n = 12) will also be interviewed. Data relating to recruitment and retention rates, and responses to questions about the intervention and travel mode in the behavioural questionnaires, will also contribute to the process evaluation.

### Sample size and power calculations

Using findings from the feasibility study, the sample size for the full-scale trial was based on an average cluster size of 8, an ICC of 0.15, and participant attrition of 25%. With a total sample size of 678 we have 80% power with a 5% significance level to detect a 15% increase in mean MVPA.

### Statistical analyses

A full plan of the primary, secondary and economic analyses will be written prior to the study statistician being unblinded. Analysis of the primary outcome (MVPA) will be based on the data from the one year follow-up, with each individual kept in the study arm to which their workplace was allocated, irrespective of whether they engaged with the intervention. This will be supplemented with a sensitivity analysis, where missing outcome data are imputed. These analyses will be as close as possible to a full intention to treat analysis.

The treatment effect will be estimated with its 95% confidence interval using regression methods, which will allow any between-workplace variation in outcome to be incorporated as a random effect (an equivalent approach to multi-level modelling), and will allow the minimisation variables to be included as covariates. In addition baseline MVPA and imbalanced baseline measures will also be included as covariates, according to a process pre-specified in the study statistical analysis plan. Linear regression will be employed in the analysis of MVPA, and ordered logistic regression in the analysis of the number of days, over the previous five working days, when walking was the major mode of travel to and from work. If there is concern that a skewed MVPA distribution is causing problems for the linear regression, this will be investigated in a sensitivity analysis of log-transformed MVPA.

This approach will be adapted and repeated for secondary outcome measures, and the post-treatment measures. Evidence of differences in the treatment effect between groups of participants defined by, in turn, age, sex, and socio-economic status will be quantified by interaction tests. Additional sub-group analyses will be pre-specified in the statistical analysis plan, but only cautious conclusions will be drawn from these as they are likely to have low statistical power. Analyses of temporal patterns of activity and of the characteristics of participant’s journeys to work will be clearly presented as exploratory in nature.

### Economic evaluation

The economic evaluation will take a broad perspective including employer, employee and public sector costs and outcomes over the one year follow-up period. We will estimate the cost of the Walk-to-Work promoter training and intervention by multiplying the time spent by trainers and employees (at training events and during workplace contacts between promoters and participating employees) by the wage rates. All expenses (e.g. materials, pedometers, room use, refreshments) involved in the intervention will be documented and costed. Absenteeism and presenteeism will be valued using wage rates and included as a cost to the employer. We will value self-reported health service use in the past month at one year follow-up using national unit costs [34]. This will provide preliminary information on any difference in health service costs. We will not assess cumulative health service costs throughout the year due to the likelihood of recall bias. We will present results as a cost-consequence study tabulating the costs and benefits of the intervention borne by employers, employees and others.

### Qualitative data analysis

All recorded interviews will be fully transcribed and anonymised. Thematic analysis [35] assisted by the Framework [36,37] method of data management, will be both inductive (focusing on key research questions) and deductive (allowing themes to emerge from the views and experiences expressed by interviewees). At least two qualitative researchers will scrutinise the qualitative data and subsequent interpretation.

### Ethical issues and research governance

The University of Bristol is the study sponsor. National Health Service (NHS) ethics approval was not required as this is not a clinical trial and does not involve patients or users of the NHS. The University of Bristol Faculty of Medicine and Dentistry Research Ethics Committee has given approval for the study (Application number: 131422 (6402)). The study will comply with the Economic and Social research Council (ESRC) framework for research ethics [38].

Full information on the research will be supplied to participating workplaces and employees. Information sheets and consent forms, written in plain English, will be included with behavioural questionnaires for all eligible employees at baseline. These will clearly state that participation in the study is voluntary. All data collected will be anonymised in any presentation of findings, and will be stored securely in line with the University’s data protection guidelines. A small amount of recompense will be supplied to employees who return accelerometers and GPS monitors in recognition of their contribution to the research. This will be handled discretely by providing individual participating employees who meet the compliance criteria with a plain envelope, with their name, containing the gift voucher and thanking them for their help with the study.

The study will be overseen by a steering group including six independent experts in the field including a statistician and a health economist. (An additional data monitoring committee was not required because this is a low risk public health intervention.) A Public Involvement Group, comprising participants from the feasibility study, was recruited at the end of the feasibility study and will be invited to meet twice a year to contribute a lay perspective to the full-scale trial.

## Discussion

The Travel to Work study is specifically concerned with walking. Compared with other forms of physical activity, walking is a popular, familiar, convenient, readily repeatable, self-reinforcing and habit-forming activity and the main option for increasing physical activity in sedentary populations [9]. There is a non-linear dose–response curve to physical activity: the greatest health benefits are achieved when the least active undertake some physical activity. In industrialised countries, higher levels of mortality and morbidity from obesity and physical inactivity-related diseases disproportionately affect those within poorer communities. Walking is the most obvious, low-cost, immediate, and normative means by which to increase physical activity and may, therefore, help to address health inequalities.

A number of high profile active travel initiatives focus on cycling [39,40]. However, walking may be perceived by employees as a more practical, cheaper and safer option: it requires no special equipment and is less likely to involve direct competition with motorised traffic for road space, and may be easily used in combination with public transport. Suggested benefits to employers of promoting walking schemes include: increased productivity, a reduction in sick leave, improved public image as a result of lowering the workplace’s carbon footprint and so contributing to any Corporate Social Responsibility commitments, and savings in providing car parking facilities [24,41-44].

The study focuses on a major public health concern: the need to increase overall rates of physical activity. In addition, the emphasis is on rigorous evaluation using objective measures to contribute to evidence based policy and practice. To our knowledge, this is the first full-scale randomised controlled trial to objectively measure (using accelerometers and GPS receivers) the effectiveness of a workplace intervention to promote walking during the commute to and from work.
